# Cultural Evolution and Perpetuation of Arbitrary Communicative Conventions in Experimental Microsocieties

**DOI:** 10.1371/journal.pone.0043807

**Published:** 2012-08-23

**Authors:** Christine A. Caldwell, Kenny Smith

**Affiliations:** 1 Psychology, School of Natural Sciences, University of Stirling, Stirling, United Kingdom; 2 School of Philosophy, Psychology, and Language Sciences, University of Edinburgh, Edinburgh, United Kingdom; Durham University, United Kingdom

## Abstract

Previous studies have shown that iconic graphical signs can evolve into symbols through repeated usage within dyads and interacting communities. Here we investigate the evolution of graphical signs over chains of participants. In these chains (or “replacement microsocieties”), membership of an interacting group changed repeatedly such that the most experienced members were continually replaced by naïve participants. Signs rapidly became symbolic, such that they were mutually incomprehensible across experienced members of different chains, and new entrants needed to learn conventionalised meanings. An objective measure of graphical complexity (perimetric complexity) showed that the signs used within the microsocieties were becoming progressively simplified over successive usage. This is the first study to show that the signs that evolve in graphical communication experiments can be transmitted to, and spontaneously adopted by, naïve participants. This provides critical support for the view that human communicative symbols could have evolved culturally from iconic representations.

## Introduction

Human language exhibits a number of unique features which account for its unparalleled expressivity and efficiency as a system of communication, the most remarkable of these being systematic compositionality (the meaning of a complex signal is [usually] a function of the meaning of its parts and the way in which they are combined [1]) and arbitrariness. Language is described as being arbitrary because words and the meanings they convey are linked by convention, rather than resemblance (although this does not mean that there is no role for iconicity in language acquisition and processing, e.g. see [2], for an enlightening re-appraisal of the role of iconicity in signed and spoken languages).

It has been proposed that these features could have emerged gradually, through a process of cultural evolution. Compositionality has been proposed to have developed from communication that was initially unsystematic and non-compositional, as a consequence of pressures for generalizability inherent in language transmission [3], [4]. Arbitrary symbols have been proposed to originate from, or through association with, more inherently meaningful (i.e. iconic) communication [5], [6]. Tomasello [6] has described the emergence of the communicative conventions of human language as a process of “drift to the arbitrary” (e.g. p. 220) over historical time, with more naturally meaningful communication, such as pantomimed gestures, as the likely origins. If these unique and universal features of human language can indeed be shown to arise spontaneously from initially unsystematic and non-arbitrary communication, then this suggests that the existence of such features is more likely the outcome of general mechanisms for learning and social cognition, in combination with repeated social transmission, rather than the consequence of an innate, specialised language faculty (see [7], [8], for elaboration of this debate).

Garrod, Fay and colleagues [5], [9], [10] have pioneered a novel experimental approach to the study of the emergence of symbolic communication, using a graphical communication task. These studies have provided valuable insights concerning the emergence of arbitrary signs from iconic communication. For example, Garrod et al. [5] showed that signs became less complex through repeated usage in a dyad, as participants established a mutual understanding about their meaning. As a consequence of this loss of complexity in the emerging graphical symbol, naïve overseers, not party to the development of the communication system, were less able to identify the concept conveyed by these signs. While the difficulty experienced by naïve overseers speaks to the arbitrary nature of the emergent graphical symbols, it proves somewhat problematic for a cultural evolution account of the origins of arbitrariness: in order to show that arbitrary communicative conventions can evolve (through cultural processes) out of iconic communication, we also need to be able to show that these arbitrary conventions, once established, can be transmitted to naïve users, and that these users will spontaneously adopt them on the basis of their value as conventionalised signs.

**Figure 1:**
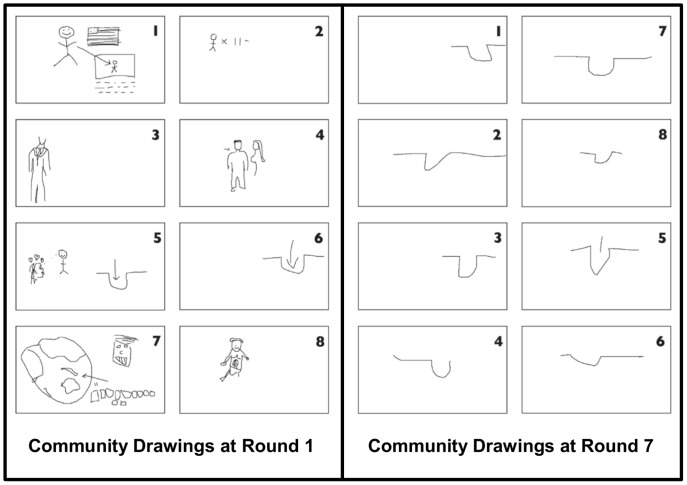
Example drawings of the concept “Brad Pitt” from one of Fay et al.’s (2010) “communities”. Individual participant numbers are given in the top right of each drawing. On each round, participants were paired with a new partner, with whom they played a total of six games, each involving the same set of list items. There were eight participants in each community. Reprinted with permission from [10, p363, Figure 2].

Fay et al.’s studies using “communities” of participants [9], [10] extend Garrod et al.’s [5] findings, and provide further illuminating insights. These communities took the form of small groups of fixed membership, whose members each interacted (pairwise) with other group members. Since participants only interacted dyadically, they lacked full knowledge of the complete history of usage of any particular sign within the broader community. Nonetheless, simplification of the graphical signs used by participants was also observed in these communities (see Fig. 1, reprinted from [10]), as it was in dyads [5]. However, since all community members played a role in negotiating the usage of a particular sign, and were likely to have contributed to its eventual form, the participants were aware of the iconic roots of the signs used by fellow community members. In line with this interpretation, new dyads working together for the first time appear to have begun by producing more iconic and elaborate signs, before progressing to the simpler versions that they had already used on previous rounds with other partners ( [10], see Fay et al.’s Fig. 4 in particular). Each individual within the community therefore does engage in some negotiation of this symbolic form, and their use of these simplified versions is apparently facilitated by an awareness of the sign’s iconic origins, as opposed to being driven purely by observation of its conventional value.

Garrod et al. [11] used the same graphical communication task to explicitly compare the roles of social coordination in dyads, versus transmission to naïve individuals in a diffusion chain paradigm, with regard to their influence on simplification of the signs. In the diffusion chains, naïve participants were exposed to already-completed drawings produced by the previous participant in their diffusion chain, before producing their own drawings which were in turn passed on, forming chains of five generations. In these non-interactive diffusion chains, drawings did not exhibit the progression towards simplicity observed in interacting pairs. Garrod et al. [11] therefore emphasised the importance of interaction in the emergence of graphical symbols. However, once again the difficulty experienced by naïve learners in this experiment appears somewhat at odds with the view that complex symbols can arise as a consequence of transmission and cultural evolution over many generations of learners.

Thus, the findings to date from the graphical communication studies of Garrod, Fay and colleagues imply that the process of simplification may be restricted to repeated horizontal interactions between users who are aware of the iconic roots of the signs they use, and who have played an active role in negotiating the simplified form. If these studies are accurately capturing the processes involved in the cultural evolution of symbols, then this would suggest that the role of cultural transmission may in fact be severely limited in explaining the emergence of symbols.

It could also be the case that experimental graphical communication studies tap into altogether different processes to those involved in real language change, in which case findings from these experiments (e.g. with regard to the necessary conditions for simplification) may not provide accurate insights into how the cultural evolution of real-world symbols might occur. Garrod et al. [5] and Fay et al. [10] have drawn attention to the similarity between their results and those from experiments on interactive spoken communication [12], [13], which also find evidence of alignment within communicating dyads, and contraction of referring expressions as a result of an interactive process of conversational grounding. Although it is unclear to what extent such conversation-level processes impact on language change beyond the dyad, some studies do indicate longer-term effects of such processes (see also [14], [15] for discussion of the role of interaction and alignment in language change). For example, naming preferences developed during one conversation have been shown to impact on interactions with a new partner [16] and simulations have shown that a population of “egocentric” agents, applying knowledge of their local interactions only, can rapidly converge on globally-shared symbolic conventions [17]. However, studies of spoken communication also show that speakers tailor their communication to the perspective of their audience, with addressee effects found in both lexical choice [13] and deletion of redundant information [18]. Graphical communication experiments may therefore simply tap into these conversation-level processes, and hence might not reflect the types of language change that occur over repeated transmission to naïve individuals.

We believe however that Garrod and Fay’s graphical communication experiments do capture important aspects of the cultural evolution of symbolic communication, and that conventionalisation and simplification can be concurrent processes, with vertical transmission leading to simplification of signs and the development of arbitrary conventions. In our design we draw on Garrod’s [5] insight regarding the importance of grounding and feedback: although our learners do not have direct experience of feedback on their *own* signs, importantly they gain observational experience of feedback (and therefore grounding), and furthermore they share this experience with their future audience.

In this study we use a graphical communication task similar to that employed by Garrod, Fay and colleagues, but adopt a “replacement microsociety” design [19], [20], [21]. With this method, participants perform the graphical communication task as a member of a small group, with experienced members of the group being removed one by one, and replaced each time with a naïve individual. This method allows researchers to investigate whether the peculiarities of particular experimentally created groups are truly cultural, in the sense of persisting in the group independent of individual membership. Such a method could therefore provide critical support for the view expressed by Garrod, Fay and colleagues [5] and Tomasello [6] regarding the cultural evolution of arbitrariness from more iconic forms of communication.

In the current study, participants took part in the task in groups of four, one participant acting as the drawer (producing drawings to convey concepts), and three acting as matchers (identifying concepts based on the drawings provided by the drawer). Each complete chain consisted of ten participants in total, and at the end of every round (during which the full set of target items were drawn once each) the most experienced member of the group was replaced by a naïve participant. This gave a total of seven rounds (see Table 1 for details). Rounds 1–6 represented the transmission phase, during which responses were made publicly and only one matcher could make a response on behalf of the group. In addition, we incorporated a test phase where the accuracy of experienced and naïve participants in identifying the meaning of signs was directly compared. The test phase occurred in the final round (round 7), during which responses were made privately on a response sheet by all matchers in the group. On this round the drawings that were shown to the matchers included some that had been completed by members of another chain, as well as some that were completed live in front of them by the seventh participant of their own microsociety.

**Table 1:** Composition of the test group over rounds of the experiment (participant numbers indicated by P[X]).

Round Number	Drawer	Matchers
1	P1	P2, P3, P4
2	P2	P3, P4, P5
3	P3	P4, P5, P6
4	P4	P5, P6, P7
5	P5	P6, P7, P8
6	P6	P7, P8, P9
7	P7	P8, P9, P10

Our primary aim was to determine whether members of microsocieties would adopt conventional signs for their communicative, rather than iconic, value (including newcomers who had played no role in their initial establishment as a communicative convention). Our design intentionally allows for the coordinated establishment of conventions, and for the natural transmission of these conventions to naïve learners. We are interested in determining whether conventions do indeed arise, resulting in exceptional recognition of their meaning by individuals who share observational experience of previous communicative acts with the sign’s producer. Crucially, in our replacement microsocieties this depends on the spontaneous adoption of these conventions by newcomers. Our participants were therefore not given any kind of explicit training on the meaning of signs used by their predecessors in the chain (as is typically provided in the classical diffusion chain design, including that used in [11]), and there was also no external instruction or incentive to reproduce signs they had observed others use. In this sense, this study is intended to allow conventions to become established and learned in a relatively naturalistic manner, whilst retaining experimental control over the prior experiences of the participants.

As noted above, Garrod et al. [5] have previously shown that “grounding” (in the form of some kind of feedback to the drawer, indicating comprehension) is crucial to the simplification of graphical signs. Our design therefore incorporated public responses by matchers, which could be observed not only by the drawer but also by the other matchers in the group. In previous studies [5], [10], [11], sign simplification has only been found in conditions involving repeated usage of the signs by particular individuals, where participants can draw on their own direct experience of effective communication. In the current study, individual participants referred to particular concepts only once, but through allowing naïve newcomers to observe responses (and therefore grounding), we predicted that simplification of signs would be possible even under such conditions of vertical transmission.

We made several predictions, each based on the assumption that the signs produced within each microsociety would exhibit a progression from relatively iconic to relatively symbolic. Firstly it was predicted that trial durations would decrease over rounds of the game. This was expected for two reasons. Firstly if drawings were becoming more symbolic we would expect that they would also be graphically less complex and therefore quicker to complete. In addition, if sign conventions were becoming established, we would also expect that drawers would exhibit less delay in their production, and matchers would have greater confidence about their meanings, both of which would contribute to shorter trial durations over rounds.

We also used an objective measure of the graphical complexity of the drawings produced (perimetric complexity, [5]). We predicted that the graphical complexity of the drawings, as measured by the perimetric complexity, would reduce over rounds, consistent with reduced iconicity and increasing arbitrariness of the signs.

It was also predicted that there would be an advantage to the most experienced matcher in the group, compared with the naïve matcher, when their accuracy was directly compared during the test phase in the final round. This prediction was again made based on the assumption that conventions would be established over rounds 1–6 and that, by the final round, the drawings would be relatively arbitrary, such that it would be difficult for a naïve person to guess their meaning, and yet easy for an individual with previous experience of the sign. It was also predicted therefore that the advantage to experienced matchers would be restricted to drawings completed by a member of their own chain, and that they would exhibit no advantage over a naïve matcher when guessing the meaning of drawings completed by a member of another chain.

## Materials and Methods

### Participants

Participants were second year Psychology undergraduates at the University of Stirling, taking part as part of a practical class on communication. In total 167 participants took part: 16 chains of 10 individuals, plus one chain of seven individuals. Drawings from all seventeen chains were analysed (since these had seven or more people and thus produced complete sets of drawings) but data on accuracy and trial times were obtained from only sixteen of these chains (i.e. those chains of exactly ten people, which were therefore more strictly comparable in later rounds).

### Ethics Statement

Ethical approval was granted by the University of Stirling Psychology Ethics Committee. All participants gave written consent to participate in the experiment.

### Apparatus

A flipchart easel was used for drawings. A fresh sheet of A4 paper was pinned to the easel on every trial, and removed at the end of that trial. The experimenter used a stopwatch to time each trial. Matchers were each provided with a complete set of laminated response cards.

### Stimulus Materials

The items to be drawn were eleven colour words. Six of these were targets, and five were filler items, included so that participants could not predict the exhaustive list for any given round. The targets (which each appeared once on every round, from rounds 1–6) were: green, grey, brown, blue, purple, pink. The fillers (two of which appeared on each round, from rounds 1–6) were: red, orange, yellow, white, black. Colour words were chosen for two main reasons. Firstly, given the replacement microsociety design, it was important that the complete list of possible targets was easy for a newcomer to grasp on entry to the test group: using colour words meant that naïve and experienced individuals were approximately equally familiar with the list items, which would not have been the case had we used a more esoteric list of concepts. Secondly, we anticipated that these relatively abstract concepts (which had to be communicated using a black pen and white paper) would be likely to be represented in different ways within different microsocieties, and that they therefore offered the potential for the development of contrasting conventions across microsocieties.

### Procedure

Volunteers were randomly assigned a participant number between 1 and 10. They were informed that they were about to take part in a graphical communication task similar to the game *Pictionary*, and that this meant they were required to communicate using only their drawings, with no speech or gestures permitted, and no numbers or letters permitted within their drawings. Both speed and accuracy were encouraged, as members of the fastest chain of participants would each win a small prize (£10 book token), and a 30 second time penalty would be added to their team’s time for every incorrect guess. Participants took part in the task in groups of four, one of whom acted as the drawer, and three of whom acted as matchers. The matchers were each provided with a full set of response cards. At the end of a round the participant playing the role of drawer was removed from the group, and the role of drawer was then taken by the most experienced matcher from the previous round. A naïve participant joined the group in the role of least experienced matcher. See Table 1 for details.

In rounds 1–6 (transmission phase) the six targets were repeated once each on every round (so every drawer was required to draw all of these items). Two of the five filler items would also appear on each round along with the six targets. The order of the items within a round was randomised. Any matcher could guess the meaning of the drawing, and to do so, they simply had to say “stop” (the only talking permitted during the task), which would stop the drawing and the timing of the trial. Responses were made in rounds 1–6 by holding up the appropriate card so that all members of the group, as well as the experimenter, could see it. On every trial the experimenter recorded which matcher said “stop” first, the trial duration (stopwatch stopped on “stop”), and whether or not the response was correct. The experimenter also informed the test group whether or not the response was correct. Incorrect responses were relatively rare (see Results section), but when they occurred the group was also informed of the intended referent.

The final round (round 7) was different from rounds 1–6 in several respects, as this was the test phase, the function of which was to compare the accuracy of naïve and experienced matchers for different drawing categories. Only targets were used (no fillers), and the matchers (P8, P9 & P10) were all required to provide their responses privately on a response sheet, guessing the meaning of: three drawings by P7 from their own chain (same microsociety); three drawings by P3 from a different chain; and three drawings by P7 from a different chain. The very first chain of participants (which was the single chain of seven, rather than ten, people, see Participants section) could not be shown drawings from another chain. However, the seventh participant from this chain did complete three drawings as did the seventh participants in every other chain, and therefore the drawings from this chain were used in the test phase for the first complete chain of ten people. Thereafter, the “different chain” drawings used were always taken from the microsociety which had been run immediately preceding the current replicate.

Those playing the role of drawer during the test phase were instructed to produce their drawings just as they would have if this round had been run in the same way as the previous rounds, i.e. requiring only one correct guess, and attempting to minimise trial durations. Accordingly, all participants involved in this round were informed that the penalty for incorrect responses would not apply to this round (to encourage drawers not to adjust their behaviour to take account of the necessity of a response by a naïve matcher). However, they were also informed that trials would be timed as before (to maintain the emphasis on speed and efficiency), and that any matcher who believed they were ready to make a response on any trial during this round should therefore say “stop”, just as in previous rounds, in order to terminate that trial.

The order of presentation of the nine drawings used in the test phase was randomised. The six drawings taken from another chain each represented the six different target words. The three drawings to be completed by participant 7 from any chain alternated between sets of three of the six target items (either: pink, purple and blue; or grey, green and brown).

## Results

### Example Drawings

**Figure 2:**
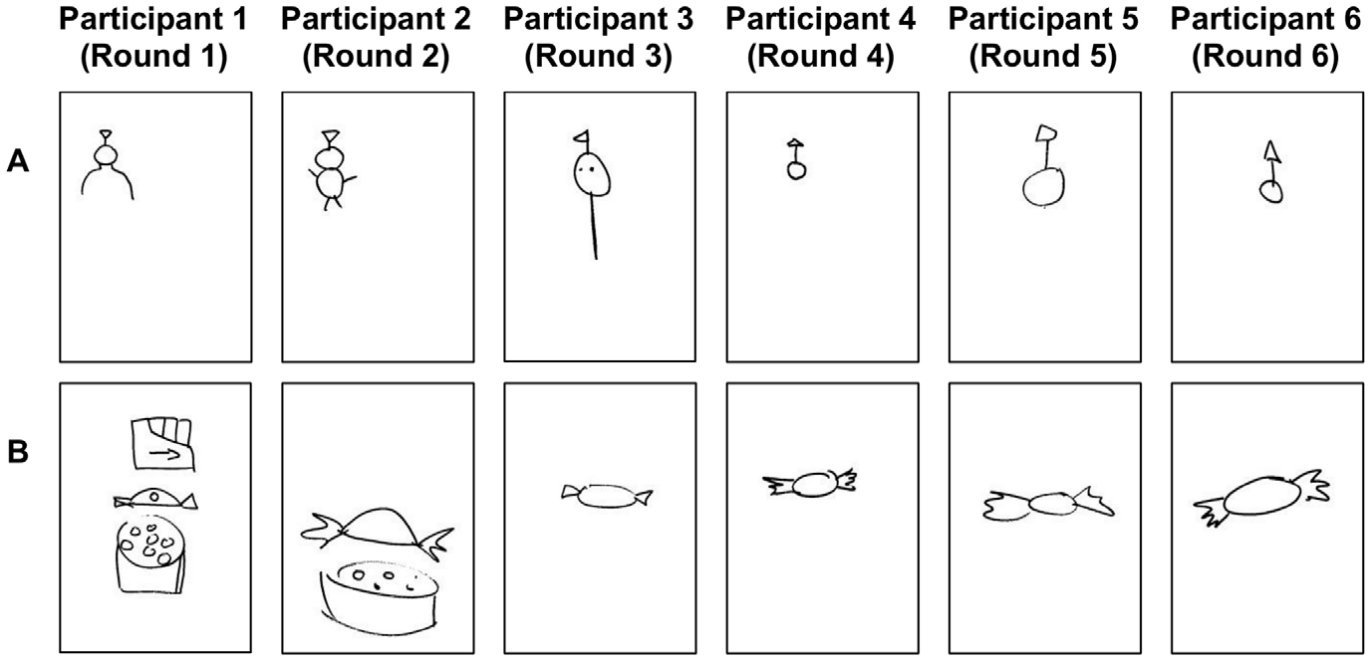
Example drawings of one of the target concepts (purple) from two microsocieties. Panels A and B each display the drawings of one chain of participants. Participant 5 joined the test group in round 2 and participant 6 joined the test group in round 3 so neither witnessed the signs produced in the initial rounds (see Table 1). Each drawing was completed on a sheet of A4 paper and they are displayed to scale here.

Figure 2 displays examples of two complete sequences of drawings of one particular target item (purple) produced by two of the microsocieties in our experiment. These particular examples illustrate relatively well the predicted effects of decreasing graphical complexity and iconicity, and they are considered in more detail in the Discussion.

### Rounds 1–6 (Transmission Phase)

The 16 complete chains together resulted in a total of 576 target trials (6 targets in each of 6 rounds for every chain). Of these, 516 (90%) were answered correctly.

From round 3 onwards all three matchers had varying levels of experience in the test group. In rounds 3 to 6 the most experienced member of any group was, overwhelmingly, the most likely to make a guess, with the least experienced member the least likely. Of the 384 target trials on these rounds, 357 (93%) were answered correctly, with 225 (63%) of these correct responses given by the most experienced matcher in the group, 114 (32%) by the next most experienced matcher, and 18 (5%) by the naïve matcher. A chi-square test showed that this was significantly different from a uniform distribution: *χ^2^* = 4180.35, *df* = 2, *p*<.001. Table 2 shows the percentage of responses which were made by each of the three matchers over rounds. This remained relatively constant between rounds 3 and 6.

**Table 2:** Mean percentage of responses (correct target trials only, *N* = 16 microsocieties) made by naïve, moderately experienced, and highly experienced matchers across rounds 1–6.

	Highly Experienced(Third Round as Matcher)		Moderately Experienced(Second Round as Matcher)		Naïve (First Round as Matcher)	
Round 1	–	–	–	–	100%	(0)
Round 2	–	–	77.71%	(21.93)	22.29%	(21.93)
Round 3	66.67%	(23.94)	30.00%	(22.64)	3.33%	(7.20)
Round 4	62.29%	(26.88)	32.29%	(25.61)	5.42%	(8.33)
Round 5	51.46%	(30.11)	35.83%	(24.11)	12.71%	(26.22)
Round 6	69.06%	(29.68)	28.33%	(30.79)	2.60%	(7.28)

Standard deviations are given in parentheses. Note that all three matchers were naïve on round 1, and on round 2 all matchers were either moderately experienced (two) or naïve (one).

**Figure 3:**
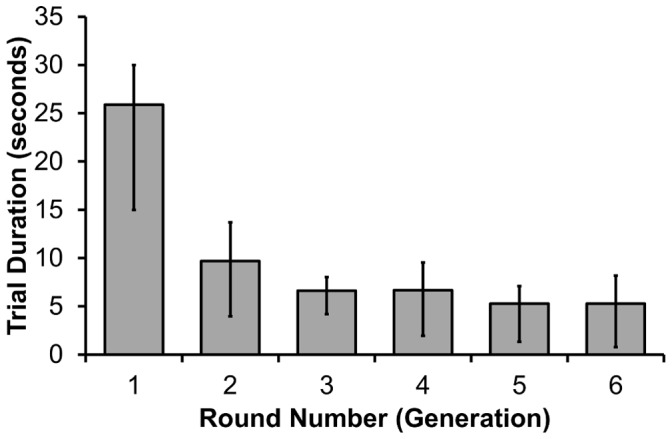
Trial duration over rounds. Median trial duration over rounds 1–6, correct target trials only. Error bars show first and third quartiles. *N* = 16 microsocieties.

With regard to the predictions stated in the introduction, it was expected that trial durations would reduce over rounds. For each chain, the average duration of correct target trials was calculated for rounds 1 to 6. These trial durations exhibited significant skewness and kurtosis and were therefore analysed using non-parametric statistics. A Friedman test showed that there were differences between rounds: *χ^2^* = 46.34, *N* = 16, *p*<.001 (Fig. 3). Pairwise Wilcoxon tests showed that trial durations were significantly higher in round 1 compared with round 2 (*Z* = 3.103, *p* = .002), and also round 2 compared with round 3 (*Z* = 2.068, *p* = .039). Further comparisons between successive rounds were non-significant: rounds 3 & 4 (*Z* = 0.534, *p* = .594); rounds 4 & 5 (*Z* = 1.914, *p* = .056); and rounds 5 & 6 (*Z* = 0.625, *p* = .532).

### Test Phase

It was predicted that experienced matchers would have an advantage over naïve matchers when guessing the meaning of drawings from their own microsociety, and that experienced matchers would also have higher accuracy when guessing the meaning of drawings from their own microsociety, compared with those from a different microsociety.

**Figure 4:**
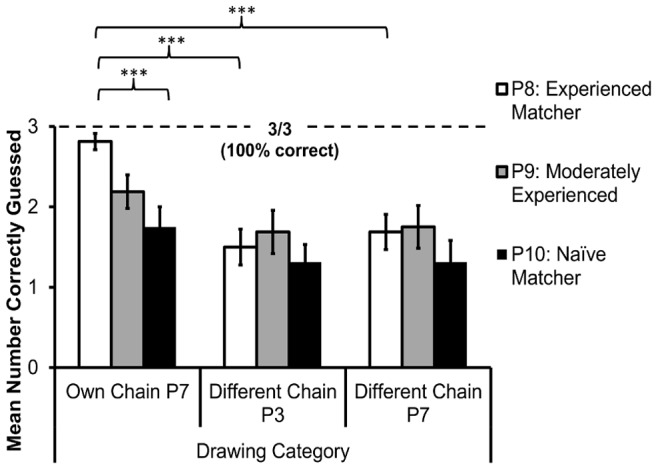
Accuracy of drawing interpretation. Mean accuracy (+/−1*SE*) of experienced (P8), moderately experienced (P9), and naïve (P10) matchers during round 7, using drawings by: P7 from their own microsociety; P3 from another microsociety; and P7 from another microsociety. Three drawings from each category were presented (so 3/3 = 100% score for that category).*** indicates *p*≤.001. *N* = 16 microsocieties.

For the test phase on the final round, the data on numbers of correct responses did not exhibit significant skewness or kurtosis, and therefore parametric statistics were used. Figure 4 displays the mean scores. A 3×3 repeated measures ANOVA was used, with level of experience (first, second and third round as a matcher) and category of drawing (completed by either: P7 from matcher’s own microsociety; P3 from another microsociety; or P7 from another microsociety) as the independent variables. Huynh-Feldt corrections have been applied where sphericity assumptions were violated.

There was a significant main effect of the level of experience of the matcher: *F*(2,30) = 10.171, *p*<.001, η^2^_p_ = .404, as well as a significant main effect of drawing category: *F*(1.484,22.260) = 5.587, *p* = .017, η^2^_p_ = .271 (Huynh-Feldt correction). There was also a significant interaction between the level of experience of the matcher and the category of drawing: *F*(4,60) = 2.871, *p* = .030, η^2^_p_ = .161.

Paired comparisons (repeated measures t-tests) were carried out between highly experienced (P8) and naïve (P10) participants within each individual drawing category, and between different drawing categories for the highly experienced (P8), and naïve (P10), participants separately. These are reported below.

#### Pairwise comparisons between P8 and P10 for the three drawing categories

Highly experienced participants scored significantly higher than naïve participants when guessing the meanings of drawings completed by a member of their own microsociety (*t* = 4.977, *df* = 15, *p*<.001, *d* = 1.574), but not for the other drawing categories. For drawings completed by P3 from another microsociety: *t* = 0.899, *df* = 15, *p* = .383, *d* = 0.225; and for drawings completed by P7 from another microsociety: *t* = 1.861, *df* = 15, *p* = .083, *d* = 0.478. This supported the predictions regarding the expertise of the experienced matchers relative to the naïve matchers.

#### Pairwise comparisons between drawing categories for P8 and P10

Highly experienced participants scored significantly higher when guessing the meanings of drawings completed by a member of their own microsociety, compared with drawings completed by P3 from another microsociety (*t* = 7.456, *df* = 15, *p*<.001, *d* = 2.408), and compared with drawings completed by P7 from another microsociety (*t* = 4.392, *df* = 15, *p* = .001, *d* = 1.149). Experienced participants showed no significant difference in accuracy when guessing the meanings of drawings completed by P3 compared with P7 from another microsociety (*t* = 0.545, *df* = 15, *p* = .594, *d* = 0.136). This was therefore consistent with the predictions regarding the expertise of the experienced matchers being specific to the drawings completed by members of their own microsociety.

Naïve participants showed no significant difference in accuracy between any drawing categories: for own microsociety drawings compared with those completed by P7 from another microsociety: *t* = 1.282, *df* = 15, *p* = .219, *d* = 0.321; for own microsociety drawings compared with those completed by P3 from another microsociety: *t* = 1.698, *df* = 15, *p* = .110, *d* = 0.427; and for drawings completed by P3 compared with P7 from another microsociety: *t* = 0, *df* = 15, *p* = 1, *d* = 0.

Significant differences are highlighted on Fig. 4 which displays the mean accuracy for the three drawing categories by experience level of participant.

### Perimetric Complexity

We also used perimetric complexity (PC) as a measure of iconicity [5], predicting that this measure would reduce over rounds within the microsocieties. Garrod et al [5] identified PC (calculated as: [Inside Perimeter + Outside Perimeter]^2/^Ink Area) as a scale-free measure of the graphical information in a picture. We calculated the PC of all drawings of target list items completed during the transmission phase (rounds 1–6) by all 17 microsocieties (see Appendix S1 for full details of how perimetric complexity was derived from the image files). This was a total of 612 drawings.

Although PC data were non-normally distributed, results were analysed using repeated measures ANOVA in order to preserve two variables (target item and generation) as repeated measures variables, to be analysed within each microsociety replicate. However, analyses were repeated on rank-transformed data (uniform distribution), as well as raw scores as a test of the robustness of the analyses with regard to distribution. Analyses performed on the non-transformed data are reported here, but results were qualitatively and quantitatively similar for both datasets. Likewise, PC values were calculated for both the raw and cleaned versions of the images (see Appendix S1) and analyses were performed on both datasets, again with results that were both qualitatively and quantitatively similar. Here we report analyses using the raw images.

**Figure 5:**
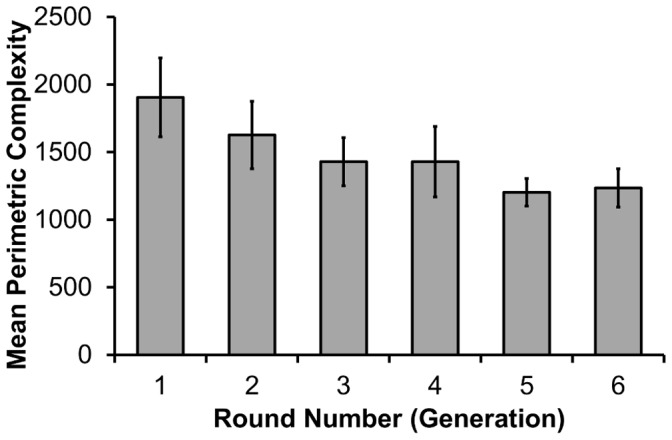
Mean perimetric complexity (+/−1*SE*) of drawings across six generations of the microsocieties (*N* = 17).

Outliers (more than 2.5 standard deviations from the mean for that particular generation and target) were trimmed. Huynh-Feldt corrections are reported where sphericity assumptions were violated. There was a significant main effect of generation, consistent with the prediction of decreasing complexity: *F*(3.795,60.717) = 3.264, *p* = .019, η^2^_p_ = .169 (Huynh-Feldt correction). There was no significant effect of target item: *F*(4.095,65.521) = 1.945, *p* = .112, η^2^_p_ = .108 (Huynh-Feldt correction), and no significant interaction between target item and generation: *F*(25,400) = 0.901, *p* = .605, η^2^_p_ = .053. Figure 5 displays the mean perimetric complexity across generations of the microsocieties.

## Discussion

The results clearly indicate that arbitrary and contrasting conventions were established within microsocieties, and that these were transmitted to naïve newcomers to the group who themselves had played no part in negotiating the usage of that particular sign. This was apparent through measures of the simplicity and efficiency of the communication (perimetric complexity and trial durations), and through comparison of the accuracy of the matchers when guessing the meanings of drawings completed by a member of their own group, compared with those completed by members of other microsocieties.

The data on accuracy generated in the test phase in the final round provide the clearest evidence that arbitrary conventions had been established and were being faithfully transmitted. In this test phase the most experienced matchers (P8) were performing virtually at ceiling level when guessing the meaning of drawings by their immediate predecessor in the chain (P7 from their own microsociety). Only three of sixteen such participants failed to correctly guess the meaning of all three of these drawings, and in each case they were only incorrect for one of the three drawings. Furthermore, this advantage to experienced matchers was not simply a function of experience with the task, since it did not extend to an advantage for guessing the meanings of drawings completed by members of different microsocieties. Also, the advantage for guessing the meaning of drawings from one’s own microsociety was not simply a function of additional information provided by the live/interactive context in which these were produced (e.g. possible movement cues while the drawing was being produced), because this advantage did not extend to the naïve matchers who were also present. Clearly therefore, the specific advantage for experienced matchers, guessing the meaning of drawings completed by a member of their own microsociety, was a function of their recognising the sign’s similarity to those produced on previous occasions. As Garrod et al. [5] have noted, “To the extent that resemblance matters at all, it is resemblance between sign and sign, not between sign and object…. Under such circumstances the sign is functioning symbolically.” (p965). For the experienced matchers in our experiment, the drawings produced by members of their own microsociety were functioning symbolically.

These results are consistent with Tomasello’s [6] view of symbolic communication as occurring through a process of “drift to the arbitrary”, as we have shown that such symbolic communication readily arises within a novel communication system, and importantly, can persist in a population. Tomasello [6] has made quite explicit that this drift to the arbitrary has only truly occurred when new users adopt a convention purely for its communicative function, naïve to its iconic roots: “individuals who are not privy to the iconic relation observe the communicative efficiency of the gesture and use it on that basis only, without any iconic motivation – at which point it has become, for these new users, arbitrary.” (p225). In this regard it is worth drawing attention to our results comparing the accuracy of responses for the meaning of drawings from round 3 and round 7 respectively. For the sets of drawings which were taken from the previous microsociety, it seems it was no easier to guess the meaning of drawings from round 3 compared with round 7, for any of the three matchers in the group. Furthermore, by round 3, trial durations had already dropped significantly compared with rounds 1 and 2, but showed little further decrease after this point. Both of these measures suggest that by round 3 the drawings had already made the transition from functioning primarily iconically (as they had to in round 1) to functioning primarily symbolically. Consistent with this, subsequent coding of the full set of drawings from this experiment by new raters (not reported in the current manuscript for reasons of brevity) has confirmed that, for drawings that were correctly guessed during the experiment, round 1 drawings are more readily interpreted than their round 3 and round 7 equivalents. Importantly, this means that participant 6 from each microsociety (who first joined the group in round 3, see Table 1) was only ever exposed to drawings which had already lost much of their complexity and iconicity.

There were several cases where it was clear from the sequence of drawings that the original iconic motivation was indeed lost by round 3. Those shown in Fig. 2 (each conveying the list item purple) represent some of the clearest examples of this. In Fig. 2a participant 1 intended to convey a children’s television character, Tinky Winky, who is purple in colour. The first drawing in this sequence is recognisable to those who know Tinky Winky, due to the shape above his head. Although the reproduction by participant 2 retains this feature, the drawing by participant 3 no longer resembles Tinky Winky specifically, but importantly *is* recognisable as a simplification of the drawings from the previous rounds. This is sufficient to retain its communicative function. The highly abstract forms produced by participants 4, 5 and 6 in this sequence suggest that they are also attempting to convey only the similarity to previous drawings, and are no longer concerned with (and quite possibly not even aware of) any residual similarity to Tinky Winky. Fig. 2b illustrates a similar case, in which a particular type of sweet (the “Purple One”, manufactured by Nestlé Quality Street) was conveyed by participant 1. The sweet is recognisable from this drawing due to its distinctive shape, as well as the inclusion of a sketch of the tin in which this sweet is usually packaged. Once again, although participant 2 retains these elements, they are lost by round 3 at which point the drawing has become a much more generic sweet, with the specific association with purple now lost.

Nonetheless, it should be noted that the iconic roots of the drawings in later rounds were in most cases still somewhat apparent. The examples illustrated in Fig. 2 were rather exceptional, albeit highly revealing. However, in the majority of cases, participants could appreciate the iconic value of the signs, especially once acquainted with the intended association through observation of others’ responses. But it was their decision to reproduce these that accounts for the advantage of the experienced matchers, and this decision was made on the basis of the established communicative value of the sign as opposed to its unambiguous interpretation. Indeed, interpretations were clearly far from unambiguous, since naïve matchers, and matchers guessing the meanings of drawings from other microsocieties were, on average, about as likely to make an incorrect response as a correct one.

The improved performance observed over the generations of the microsocieties also represents an example of cumulative cultural evolution [22]. This improvement in performance is clear from both the reduction in trial durations (which was the goal measure as conveyed explicitly to the participants), and also the reduction in perimetric complexity, which provides an excellent proxy measure for the effort involved in communicating the concept. Later participants were therefore able to benefit from, and incrementally modify, the behaviours they observed in their predecessors.

In previous studies of cumulative culture in experimental replacement microsocieties, which used building tasks with objective goal measures (height and distance), Caldwell and Millen [23] found some evidence of between-group variation in designs, but also evidence of independent convergence on similar (effective) designs towards the ends of chains. In [23], participants’ choices were shaped by the demands of the tasks, and since these were common across microsocieties, similar selection pressures were exerted on the cultural evolution of the designs. However, the development, and continued transmission, of arbitrary conventions found in the current experiment illustrates that cumulative cultural evolution need not always result in convergence on similar adaptive solutions across populations. Communicative conventions are of course arbitrary in the sense that their form is not dictated by their meaning, but they are nonetheless highly functional in that they permit efficient communication, assuming others are familiar with them. Consequently cumulative cultural evolution can, for certain behaviours, simultaneously result in both increased efficiency of the behaviours concerned, and sustained variation between groups which can persist over multiple transmission events.

Returning to the theoretical issues raised in the introduction, this study also illustrates that conventionalisation and simplification can be concurrent processes, as successive simplification of signs occurred over experimental generations of new sign users, rather than just within particular individuals’ usage histories. This demonstration therefore provides additional support for the argument that the complex symbolic representations of language could have arisen as a consequence of cultural transmission and evolution facilitated by general purpose cognitive mechanisms, rather than requiring a specialised language faculty [24]. Previous research [5], [11] has provided valuable insights into the necessary conditions for the emergence of symbolic communication, but based on these previous studies it was unclear how the emergent symbols might be transmitted, and how adoption by naïve users might impact on their form. A cultural evolution viewpoint on the emergence of symbols over multiple generations (such as the hypothesis of the “drift to the arbitrary”, [6]) essentially entails that symbols arise in the absence of any explicit intent on the part of particular individuals, with variation between users as the primary driver of simplification, overshadowing any simplification that might occur within a single individual’s history of usage.

Our study suggests that observational experience of grounding, shared with one’s future audience, is sufficient to allow conventionalisation and successive simplification of signs over a chain of participants. It remains to be seen to what extent recognition of mutual knowledge with the audience is necessary for this to occur [17], and indeed whether the observational experience of feedback must be quite as explicit as it was in our study, with sign-meaning mappings effectively modelled for newcomers.

## Supporting Information

Appendix S1 Calculation of perimetric complexity.
(DOCX)
