# Supplementary material for: Cultural Evolution and Perpetuation of Arbitrary Communicative Conventions in Experimental Microsocieties
Source: PLoS One. 2012 Aug 23;7(8):e43807. doi: 10.1371/journal.pone.0043807 (PMC3426541; doi:10.1371/journal.pone.0043807)
Supplement: Appendix S1 — Calculation of perimetric complexity. (DOCX) [file pone.0043807.s001.docx]

**Appendix S1:** Calculation of perimetric complexity

Perimetric complexity was calculated as follows:

1. Each A4 drawing was scanned to PDF at 300dpi.
2. Scanned images were then cropped to contain all participant-produced lines but eliminate any experimenter annotations or edge-of-paper artifacts arising during scanning.
3. The resulting cropped PDF was converted to a plain PBM image using ImageMagick version 6.4.1 (command: convert FILENAME.pdf +dither -colors 2 -colorspace gray -normalize -compress none FILENAME.pbm).
4. The resulting PBM was cleaned using a custom program to remove isolated pixels: any pixel with a zero or one neighboring pixels of the same value was flipped to the opposite value, with this process being applied iteratively until no pixels were flipped.
5. Perimeter images were then calculated for both raw and cleaned versions via a custom program using the two-stage method provided by Pelli et al (2006).
   1. Generate a one-pixel wide perimeter image by ORing the original image with all eight possible translations of the original generated by shifting by one pixel, and then bit clear with the original image.
   2. Generate a three-pixel wide perimeter by ORing the one-pixel wide perimeter with the four translations of itself obtained by shifting left, right, up or down; 6). The three-pixel wide perimeter image was used in conjunction with the original to calculate the perimetric complexity of the image. The ink area of the three-pixel wide perimeter image was divided by 3 to correct for the use of a thickened perimeter image.

Pelli DG, Burns CW, Farrell B, Moore DC (2006) Feature detection and letter identification. Vision Res 46: 4646–4674.
